# Current Diagnosis, Treatment and Clinical Challenges in the Management of Lipodystrophy Syndromes in Children and Young People

**DOI:** 10.4274/jcrpe.galenos.2019.2019.0124

**Published:** 2020-03-19

**Authors:** Samim Özen, Barış Akıncı, Elif A. Oral

**Affiliations:** 1 Ege University Faculty of Medicine, Department of Pediatric Endocrinology, İzmir, Turkey; 2 Dokuz Eylül University Faculty of Medicine, Department of Internal Medicine, Division of Endocrinology and Metabolism, İzmir, Turkey; 3 University of Michigan Medical School, Department of Medicine, and Brehm Center for Diabetes, Division of Metabolism, Endocrinology, and Diabetes, Michigan, USA

**Keywords:** Lipodystrophy, childhood, adolescence, progeria, metreleptin

## Abstract

Lipodystrophy is a heterogeneous group of disorders characterized by lack of body fat in characteristic patterns, which can be genetic or acquired. Lipodystrophy is associated with insulin resistance that can develop in childhood and adolescence, and usually leads to severe metabolic complications. Diabetes mellitus, hypertriglyceridemia, and hepatic steatosis ordinarily develop in these patients, and most girls suffer from menstrual abnormalities. Severe complications develop at a relatively young age, which include episodes of acute pancreatitis, renal failure, cirrhosis, and complex cardiovascular diseases, and all of these are associated with serious morbidity. Treatment of lipodystrophy consists of medical nutritional therapy, exercise, and the use of anti-hyperglycemic and lipid-lowering agents. New treatment modalities, such as metreleptin replacement, promise much in the treatment of metabolic abnormalities secondary to lipodystrophy. Current challenges in the management of lipodystrophy in children and adolescents include, but are not limited to: (1) establishing specialized centers with experience in providing care for lipodystrophy presenting in childhood and adolescence; (2) optimizing algorithms that can provide some guidance for the use of standard and novel therapies to ensure adequate metabolic control and to prevent complications; (3) educating patients and their parents about lipodystrophy management; (4) improving patient adherence to chronic therapies; (5) reducing barriers to access to novel treatments; and (5) improving the quality of life of these patients and their families.

## Introduction

Lipodystrophy is the general term for a heterogeneous group of disorders characterized by near total [generalized lipodystrophy (GL)] or partial [partial lipodystrophy (PL)] lack of body fat (1). Lipodystrophy can be genetic or acquired. Congenital GL (CGL), familial PL (FPLD), acquired GL (AGL), and acquired PL (APL) make up the four major categories of lipodystrophy in clinical practice, although there are several others such as progeria associated lipodystrophy, auto-inflammatory syndromes, and complex syndromes associated with lipodystrophy (1,2,3,4,5). The current classification schema, which is based on clinical presentation, may change as our understanding of the disease processes improve. In this paper, we will focus on presentation of various forms of lipodystrophy during childhood and adolescence and then review the general clinical approach for these patients.

## Types of Lipodystrophy in Children and Young People

### Genetic Lipodystrophy Syndromes

### Congenital Generalized Lipodystrophy

CGL (Berardinelli-Seip syndrome) is the most common lipodystrophy subtype in infancy and early childhood, while the incidence of FPLD increases close to puberty (3,4,5,6). CGL cases made up almost half (519 of 1141) of pediatric patients with non-human immunodeficiency virus lipodystrophy identified by a recent systematic review of a total of 351 studies (6). CGL is a rare disorder with autosomal recessive inheritance, in which patients are born with near total lack of body fat. These patients have a remarkable phenotype characterized by near total absence of adipose tissue, muscular overdevelopment and prominent subcutaneous veins, which can be noticed either at birth or in the first year of life (7). The prevalence of CGL is uncertain, but it has been estimated at approximately 1:10 million (8). A recent study reported an estimated prevalence of 0.23 cases/million for diagnosed GL (9). However, CGL has a higher prevalence in certain parts of the world as a result of consanguineous marriages. We reported an estimated CGL prevalence of 1:2 million in Turkey, considerably higher than in other reports but still quite rare (10).

There are four major subtypes of CGL based on mechanism:

CGL1 [Online Mendelian Inheritance in Man (OMIM) #608594] is caused by pathogenic variants of the 1 acylglycerol 3-phosphate acyltransferase β2 (*AGPAT2*) gene (11). The AGPAT2 enzyme converts lysophosphatidic acid into phosphatidic acid, a critical step in triglyceride synthesis (12). Homozygous pathogenic variants that eliminate enzyme activity have been demonstrated in the majority of CGL1 cases. Compound heterozygous or homozygous pathogenic variants with low levels of *in vitro* enzyme activity have also been reported (7,13). Although patients with CGL1 lack metabolically active adipose tissue, the preservation of residual mechanical adipose tissue in the palms, soles, scalp, orbital and periarticular regions and the perineum is clinically apparent (14,15).

CGL2 (OMIM #269700) is caused by pathogenic variants of the Berardinelli-Seip congenital lipodystrophy 2 (*BSCL2*) gene (16), which encodes the transmembrane protein seipin. This protein is involved in the fusion of small lipid droplets in adipocytes, as well as in the differentiation of adipocytes (17). The majority of identified variants have been classified as null pathogenic variants, based on functional investigations, which lead to severe disruption of the protein. Missense pathogenic variants have also been reported (7,13,18).

Pathogenic variants of the caveolin 1 (*CAV1*) gene, which encodes caveolin 1, a principal component of the caveolae, causes CGL3 (OMIM #612526). A homozygous nonsense pathogenic variant in *CAV1* was reported in a patient with CGL from Brazil (19). Magnetic resonance imaging of the proband confirmed the absence of metabolically active adipose tissue, while bone marrow adipose tissue was preserved (19). Heterozygous *CAV1* pathogenic variants have also been associated with PL (20).

CGL4 (OMIM #613327) is caused by homozygous or compound heterozygous pathogenic variants in the polymerase 1 and transcript release factor (*PTRF*, or cavin-1) gene (21). PTRF regulates the expression of caveolins 1 and 3. Cavin-1 plays a major role in the formation of caveolae and caveolin stabilization through interaction with the cellular cytoskeleton. Cavin-1 regulates adipocyte differentiation and it is a determining factor in the capacity of adipose tissue to expand (22). CGL3 and CGL4 have distinct clinical characteristics (Table 1) (23,24,25).

In addition to these four major groups of CGL, patients with GL associated with Lamin A/C (*LMNA*) p.T10I (26), and biallelic peroxisome proliferator activated receptor gamma (*PPARG*) (27) pathogenic variants have been reported. Patients with biallelic loss-of-function pathogenic variants in phosphate cytidylyltransferase 1 alpha (*PCYT1A*) gene were reported to have a severe PL phenotype (28), and potentially can be classified in the CGL category.

Table 1 summarizes subtypes of CGL.

## Familial Partial Lipodystrophy

FPLD is a subtype of lipodystrophy with a genetic background, which in adults is more common than any other subtype of lipodystrophy (4,8,9,29). FPLD exhibits a typical fat tissue distribution. Phenotypic features are more prominent in females. Loss of adipose tissue is predominantly observed in the upper and lower extremities. Patients may exhibit accumulation of fat in certain areas such as the face and neck, and perineal and intra-abdominal depots (2,4,30,31,32). A Cushingoid-appearance can be observed, due to thin limbs, facial fat accumulation and increased fat in the dorsocervical region resembling a ‘buffalo hump’ (5). The partial loss of fat may be apparent in early life, but typically becomes more pronounced over time in FPLD, and most patients start to lose adipose tissue after puberty (30). For this reason, it is difficult to recognize these patients in childhood. However, a few patients with FPLD have been reported from pediatric endocrinology practices (6).

Most FPLD subtypes are inherited in an autosomal dominant (AD) manner. However, recent evidence suggests that a large group of patients with FPLD1, also known as Koberling-type FPLD (OMIM #608600), follow a polygenic inheritance pattern (33,34,35). FPLD2, the Dunnigan variety, (OMIM #151660), is caused by AD pathogenic variants in the *LMNA* gene (36), which encodes nuclear lamins A and C (36,37). *LMNA* R482W and R482Q are the most common pathogenic variants (38,39). FPLD3 (OMIM #604367) is caused by AD pathogenic variants in the peroxisome proliferator-activated receptor gamma gene (*PPARG*) on chromosome 3p25 (40,41). PPARG plays a major role in the regulation of adiposity differentiation (40). Other subtypes of FPLD are rare. A recent review of these subtypes is available (1). The subtypes of FPLD are presented in Table 2.

## Acquired Lipodystrophy Syndromes

### Acquired Generalized Lipodystrophy, Lawrence Syndrome

AGL affects the whole body and causes generalized fat loss. Patients develop typical metabolic complications of lipodystrophy including severe insulin resistance, diabetes, hypertriglyceridemia and non-alcoholic steatohepatitis (NASH) (42). The clinical phenotype is very similar to that of CGL. However, patients with AGL are born with normal fat tissue. Loss of adipose tissue typically begins in childhood or adolescence. Marked phenotypic features occur over differing lengths of time, from a few weeks to a year. The cause of the disease is still unknown. The disease coincides with other autoimmune diseases such as juvenile dermatomyositis, type 1 diabetes and autoimmune hepatitis. AGL is associated with panniculitis in some patients. Complement abnormalities may also be present (8,42,43).

### Acquired Partial Lipodystrophy, Barraquer-Simons Syndrome

APL (OMIM #608709) is characterized by the loss of fat from the face, neck, arms, chest and abdomen with preservation of lower extremity fat. Clinical onset typically occurs in childhood or adolescence and females predominate at a ratio of 4:1. Loss of fat first manifests in the face, and gradually progresses to the upper extremities, thorax and upper abdomen, symmetrically and in a cephalocaudal fashion. Excessive deposition of fat may be observed in the lower limbs (44). The etiology of APL remains unknown, however, there is a link to autoimmune abnormalities and coincidental autoimmune conditions can be observed. APL is associated with low complement factor 3 (C3) levels and membranoproliferative glomerulonephritis in some patients, which may cause end stage renal disease in some patients (44,45,46). Drusen deposition in the macula has also been reported.

### Progeroid Disorders and Other Rare Genetic Lipodystrophy Syndromes

Lipodystrophy is a part of the clinical picture in many progeroid syndromes (1,47,48). There are also several other complex syndromes associated with lipodystrophy (1). The characteristics of these syndromes are presented in Table 3.

### Other Causes of Acquired Lipodystrophy

The development of lipodystrophy after whole body irradiation in preparation for bone marrow transplant (49,50,51,52,53,54), following cranial irradiation and as a result of hypothalamic tumors (55) has been described and is a clinically neglected cause of lipodystrophy. When children have aggressive treatments for childhood cancers, they should be assessed for signs of fat loss and ensuing metabolic abnormalities. In a young child presenting with lipodystrophy, it is very important to consider the possibility of central nervous system tumors, especially when the clinical presentation does not fit with AGL. Other forms of cancer therapy, such as checkpoint inhibitors, may lead to fat loss to varying extents (56,57). These unusual forms of acquired lipodystrophy will be reviewed in a specific review in the near future.

## Clinical Features of Lipodystrophy

### Leptin Levels

GL is characterized by very low or undetectable levels of leptin. However, in PL leptin concentrations may range from low to normal (58). Several studies have suggested that baseline serum leptin measurement may assist physicians to identify PL patients who are more likely to benefit from leptin replacement (59,60). Leptin concentrations correlate with fat mass, and females have higher leptin concentrations than men in adulthood (61). However, it is challenging to interpret leptin levels in infancy, childhood and adolescence. Girls generally have higher leptin levels after the completion of puberty, as might be expected given the difference in concentrations seen in adults of different genders (62,63). Prepubertal levels of leptin are similar in both sexes, although concentrations fluctuate during infancy (62,64). Younger infants have higher leptin levels than older infants presumably secondary to an initial increase in breast milk leptin from colostrum to mature milk, which is followed by a decreasing trend during the first months of lactation and a subsequent increase during the late period of lactation (65,66,67). Leptin concentrations correlate with age in prepubertal girls and boys and increase in both boys and girls during early puberty. In boys, this early pubertal leptin peak is followed by a decrease in leptin concentrations several years after the rise in serum testosterone levels. However, in girls, leptin concentrations continue to increase during puberty in parallel with increasing levels of estrogen. The concentration of leptin peaks in mid-puberty and maintains this plateau level thereafter (62). Thus, clinicians should pay attention to many factors when interpreting leptin concentrations. Serum leptin measurement may help clinicians with the management of lipodystrophy, but it should not be considered as a reliable tool to diagnose or rule out lipodystrophy. In addition to leptin, adiponectin concentrations are helpful in certain patients. A relatively high concentration of adiponectin is a distinguished characteristic of CGL2, although serum leptin is extremely low in all subtypes of CGL (10,68).

### Metabolic Abnormalities

Patients with lipodystrophy may develop metabolic abnormalities associated with insulin resistance before adulthood, which are more severe and have an earlier onset in GL (3,31,43,69). These metabolic abnormalities include, but are not limited to, diabetes, hypertriglyceridemia, hepatic steatosis, and NASH (3). Most females with lipodystrophy suffer from polycystic ovary syndrome (PCOS). Some of the factors determining the severity of metabolic abnormalities are the degree of adipose tissue loss, type of lipodystrophy, age, and sex. However, the severity of metabolic abnormalities may vary even among subjects with the same genetic pathogenic variant (30,32,70,71).

Hyperphagia usually develops due to severe leptin deficiency in early childhood in subjects with CGL (7). Accelerated linear growth, advanced bone age and signs indicative of acromegaly, such as enlargement of the hands, feet and jaw, may be observed (3). Cystic bone lesions are frequently noted in CGL1 patients (10,72,73). Hyperinsulinemia and severe insulin resistance develop in the majority of patients with CGL due to near total adipose tissue loss and leptin deficiency (10,74). Acanthosis nigricans (AN), a clinical marker of insulin resistance, can be observed in body folds such as the axilla and neck during childhood, with a strong likelihood of a further increase after puberty (3,8). Approximately 45% of patients with CGL develop diabetes mellitus before puberty (7,10). The treatment of diabetes is challenging, and high-doses of insulin (>100 units/day) may be required. Although most patients are poorly controlled, diabetic ketoacidosis rarely develops, but has been reported, probably as a result of severe hyperinsulinemia and lack of fat tissue (4). Hypertriglyceridemia usually develops in childhood. Eruptive xanthomas and recurrent episodes of pancreatitis caused by severe hypertriglyceridemia may emerge after puberty (3). Hepatic disease is more aggressive in patients with CGL2 (10). Hepatomegaly is remarkable at very young ages (3,10). Patients may develop cirrhosis during childhood (10). PCOS, hyperandrogenism, and menstrual irregularity are common in adolescent girls (75).

AN, metabolic abnormalities associated with insulin resistance and hepatic steatosis may be observed in young people with FPLD (31), albeit milder in presentation than in CGL. Hypertriglyceridemia is common and can be severe. Episodes of acute pancreatitis may be observed. Cardiomyopathy and myopathy, as well as features resembling muscular dystrophy, may be detected in patients with FPLD2 (8,31,59). In contrast to other forms of lipodystrophy, in APL metabolic complications have rarely been reported (44). However, our previous observations suggested that a subgroup of patients with APL developed metabolic abnormalities associated with insulin resistance, which can also be progressive in some cases. Although the majority of these patients were adults, a few of them developed metabolic abnormalities in their younger years (46).

## Treatment of Lipodystrophy in Children and Young People

### General Guidelines

Although there is no curative treatment for lipodystrophy syndromes, early diagnosis of these patients may prevent mortality or serious morbidity. The aim of medical treatment is to correct metabolic abnormalities associated with lipodystrophy and prevent end-organ complications. Medical nutrition therapy (MNT) and exercise are important tools in the management of patients with lipodystrophy, although it is not easy for patients and families to comply with these therapies. Hyperphagia due to leptin deficiency is a serious problem in these children. However, weight increase is only part of the clinical problem. Since overeating exacerbates hepatic steatosis, diabetes and hyperlipidemia, particularly in babies and children, dietary fat intake should be reduced, and fat should be predominantly taken in the form of cis-monounsaturated fats and long chain omega-3 fatty acids. Medium chain triglycerides in infant formulas may be helpful to reduce triglyceride levels. In patients developing acute pancreatitis secondary to hypertriglyceridemia the amount of dietary fat should be severely restricted. Patients with diabetes also have to balance their intake of carbohydrates (4). Exercise may improve metabolic parameters in patients with lipodystrophy. Sedentary time should be reduced as much as possible, with a focus on minimizing time spent on computer and television. Physical activity should be advised, unless contraindicated, and a detailed cardiac examination should be performed before advising an exercise plan. Special attention should be given to patients with CGL4, FPLD2 and progeroid syndromes. CGL patients with lytic bone lesions and also patients with hepatosplenomegaly should avoid contact sports (1,4).

The use of lipid lowering drugs should be considered in children and adolescents with lipodystrophy when diet and exercise fail to control triglyceride levels. Fibrates are commonly used in children with very high triglyceride levels who are at risk for pancreatitis (76). Omega-3 fish oil therapy may also help to reduce triglyceride levels (76,77). Although fibrates, alone and in combination with statins, have been shown to effectively reduce triglyceride levels in adults, data are very sparse in children (6,77). Physicians should pay attention to safety measures while using fibrates for hypertriglyceridemia in children. Liver enzymes should be monitored. The risk of rhabdomyolysis should be kept in mind.

Metformin is the first choice to treat insulin resistant diabetes in children, in addition to MNT and lifestyle modification. The American Food and Drug Administration (FDA) approved the use of metformin for pediatric patients 10 years of age or older, noting that the safety and effectiveness of metformin have been shown in pediatric patients ages 10 to 16 years (78). Lipodystrophy patients with diabetes usually require insulin injections in combination with metformin (6). Insulin doses required to cause an effect may need to be very high, and patients may need to use concentrated forms of insulin (4).

Most patients with lipodystrophy desire a better cosmetic appearance. Patients with lipodystrophy may consider having cosmetic surgery, which may help them feel better about their physical appearance and may offer an improved quality of life. Excess unwanted localized fat can be removed from several locations that include the chin, buffalo hump, and vulvar region by liposuction or surgical excision. Lipoatrophic areas may also benefit from autologous adipose tissue transplantation, facial reconstruction and implants (1).

## Metreleptin Therapy

Recombinant human leptin (metreleptin) therapy can be used to minimize and prevent complications of lipodystrophy (79). Severe hypoleptinemia causes hyperphagia and exacerbates metabolic complications associated with insulin resistance in patients with lipodystrophy (80). Several long-term studies have shown beneficial effects of metreleptin in GL with severely low serum leptin levels (81,82,83,84). Metreleptin therapy has been associated with a significant reduction in triglyceride and hemoglobin A1c (HbA1c) levels, and improvements in appetite control, insulin sensitivity and hepatic steatosis (82,85,86,87,88,89).

The metabolic effects of metreleptin are remarkable in pediatric patients with lipodystrophy. In the largest report of the efficacy of metreleptin in pediatric patients with lipodystrophy to date, Brown et al (85) showed a reduction of HbA1c from a mean level of 8.3% to 6.5%. Triglyceride levels significantly decreased from 374 mg/dL to 189 mg/dL. The benefit was more prominent in adolescents (from 9.6% to 7.1% for HbA1c, and from 556 mg/dL to 226 mg/dL for triglycerides), presumably because of the presence of a more severe disease at baseline. Insulin sensitivity improved, and half of the patients who were on insulin at baseline were able to discontinue insulin treatment after metreleptin. The levels of liver enzymes decreased, and liver histology improved in a subset of patients who underwent before and after treatment liver biopsies. Metreleptin therapy was also associated with partial normalization of rapid growth in CGL, and improvements in pubertal development. The average dose of metreleptin was 0.082 mg/kg/day (range: 0.04 to 0.19 mg/kg/day; absolute dose: 4.1 mg/day). The dose per kg was nonsignificantly higher in adolescents. Patients with FPLD were treated with higher doses of metreleptin compared to those with GL.

Metreleptin was approved by the FDA in 2014 for treatment of adult and pediatric patients with GL to treat metabolic complications of lipodystrophy, as an adjunct to diet and lifestyle modifications (90,91). Although metreleptin therapy resulted in heterogeneous improvements in patients with PL, in a subset probably consisting of PL patients with low leptin levels, metreleptin is likely to be beneficial (59,60). Although the FDA has not yet approved metreleptin for treatment of PL, the European Medicines Agency’s (EMA) Committee for Medicinal Products for Human Use (CHMP) has approved metreleptin in patients with PL in whom standard treatments have failed to achieve adequate metabolic control (92). There is no age limit for treatment in the US, and it has been used in infants as young as six months of age (85). However, the EMA CHMP’s recent positive opinion includes authorization for metreleptin only in children two years of age and above with GL, and in children 12 years of age and above with PL (93). In Turkey, metreleptin therapy is currently available only for GL patients for whom standard treatments have failed to control HbA1c and triglyceride levels (94).

The generally recommended starting dose of metreleptin is 5 mg/day in females greater than 40 kg, 2.5 mg/day in males greater than 40 kg, and 0.06 mg/kg/day (0.012 mL/kg) in males and females less than or equal to 40 kg (91). The dose should be kg based in children, and the physicians should keep in mind that most children will require increasing per kg doses, especially as they reach puberty. However, the dose should be adjusted based on clinical response, and tolerability issues should also be borne in mind including excessive weight loss in pediatric patients. Metreleptin can be given once daily at any time of day regardless of meals (4,5). The most common side-effects are hypoglycemia and injection site reactions, such as erythema and/or urticarial (91). Metreleptin therapy has been shown to have beneficial effects on kidney function (95). However, a few patients have been described with worsening of proteinuria on metreleptin (91). This observation needs to be confirmed empirically as the disease progression itself can cause worsening proteinuria.

Two important issues which led to a “black box” warning in the US need to be specifically discussed. These are the development of neutralizing antibody and T-cell lymphoma. Anti-metreleptin antibodies were reported in a considerable number of patients with lipodystrophy on metreleptin. However, antibodies with *in vivo* neutralizing activity have only been detected in a small number of patients (96). T-cell lymphoma has been reported in a few patients with AGL treated with metreleptin (97,98). Immune dysfunction is a feature of the natural history in patients with AGL (42,44). To date no lymphoma development has been reported in patients with CGL or FPLD treated with metreleptin. Current evidence suggests that lymphoma development in patients with AGL may be associated with the natural history of the disease rather than being a treatment effect associated with metreleptin.

## Challenges in the Management of Lipodystrophy in Childhood and Adolescence

The needs of pediatric patients are different to those of adults and there are several challenges specific to children and young people in the management of lipodystrophy. Crying gives the baby a way to call for help when he/she is hungry or uncomfortable. Babies with lipodystrophy feel hungry all the time because of leptin deficiency. Appetite control is almost impossible in GL especially during active growth without getting help from metreleptin therapy. Even on metreleptin, patients and parents struggle to decide on the right amount of food to consume. Metreleptin causes weight loss in most patients. Parents become stressed when they witness their children losing weight on metreleptin, especially as they already look very thin because of the lipodystrophy.

Small children may have difficulties with verbalizing symptoms, such as abdominal pain caused by acute pancreatitis, symptoms of hyper- or hypoglycemia, muscle symptoms and infections. It may also be difficult to explain to children and adolescents why metabolic control is critical in lipodystrophy. The need for different types of therapies, and tasks such as glucose monitoring, routine blood sampling and regular hospital visits, being careful with what type of food is eaten and how much food is eaten given the associated hyperphagia is overwhelming for many of them. Children with lipodystrophy may need multiple injectable treatments including both insulin and metreleptin. Even though metreleptin therapy may allows the reduction in frequency or even the discontinuation of insulin injections metreleptin is still an injectable agent. In addition the lack of subcutaneous tissue makes the injection technique more challenging in lipodystrophy. It should be also noted that the relatively large injection volume would be another issue with metreleptin injections. It may prove difficult to get an active child to accept injections and self-monitoring of blood glucose at home.

Physical appearance is a big problem for adolescents with lipodystrophy. They may be worried about being with their friends and in new social environments. Anger and temper outbursts are common. They may also have specific fears associated with the subtype of lipodystrophy.

To provide timely diagnosis and improve the delivery and quality of care, specialized centers for lipodystrophy for affected children and adolescents should be established. Optimizing management algorithms for children and young people can provide guidance for the use of standard and novel therapies to ensure adequate metabolic control and to prevent complications of lipodystrophy. These centers would develop more experience and thus be able to provide better and more efficient education for patients and their parents about lipodystrophy and its management. This should result in improved adherence to therapies and quality of life for these patients and their families. Cost of treatment remains the biggest barrier to access to novel treatments, such as metreleptin. However, the recent EMA approval of metreleptin therapy is a promising development for lipodystrophy patients in Europe and elsewhere in the world where the recommendations of EMA are adopted by the local authorities. It is important to state that regulatory authority approval does not guarantee access, if local health care coverage programs do not include therapies for rare diseases in their formularies. If metreleptin therapy is not an option in a specific country, or for a patient’s plan, access to metreleptin can potentially be established through compassionate use programs and other regulatory mechanisms.

## Conclusion

Lipodystrophy syndromes are a heterogeneous group of disorders, characterized by the lack of adipose tissue, and is associated with severe insulin resistance that usually results in metabolic abnormalities leading to serious morbidity and increased mortality. Although there is no definitive cure for lipodystrophy, patients may benefit from an early diagnosis made in childhood, which in turn may improve lipodystrophy outcomes by providing care at the earliest stage possible. Effective strategies should be developed to overcome challenges in the management of lipodystrophy in children and young people.

## Figures and Tables

**Table 1:**
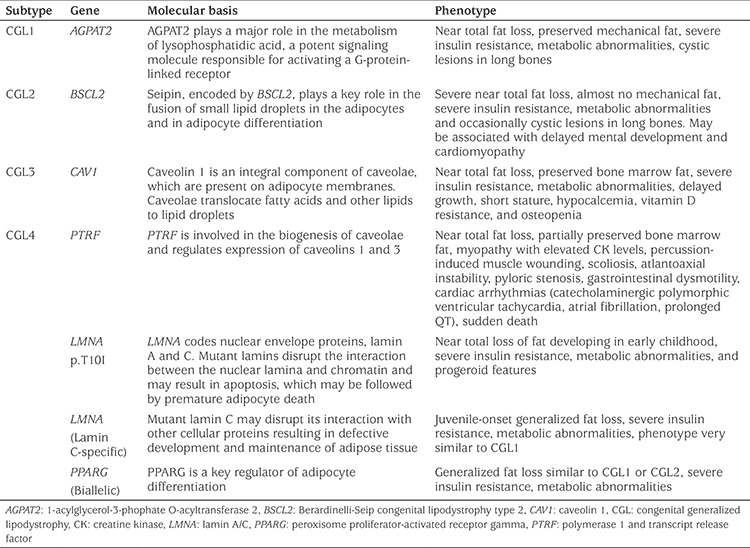
Subtypes of congenital generalized lipodystrophy, and their genetic and clinical characteristics

**Table 2:**
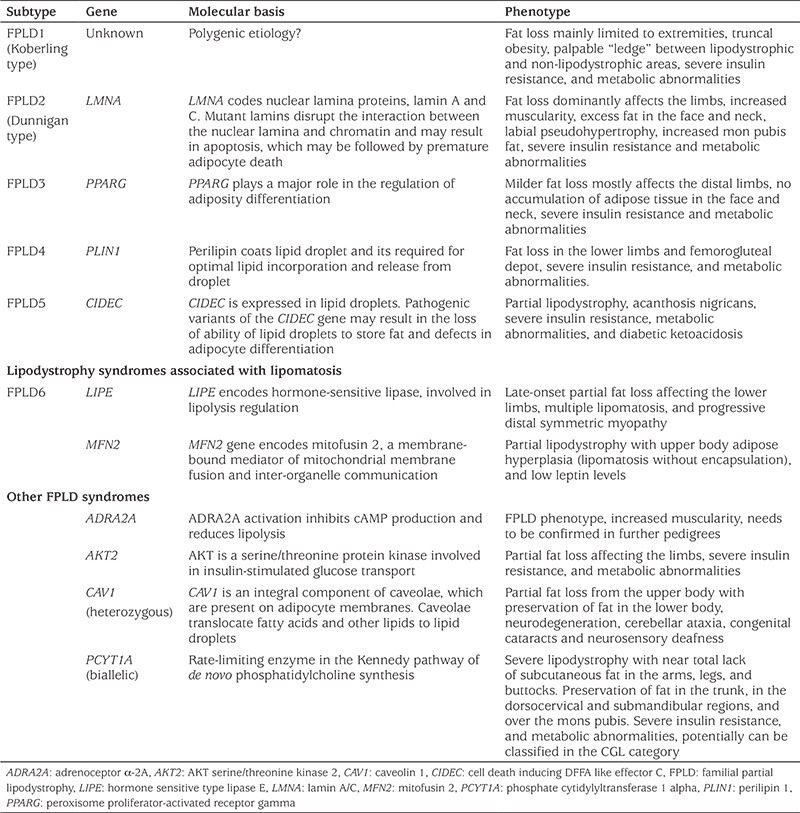
Subtypes of familial partial lipodystrophy and their genetic and clinical characteristics

**Table 3:**
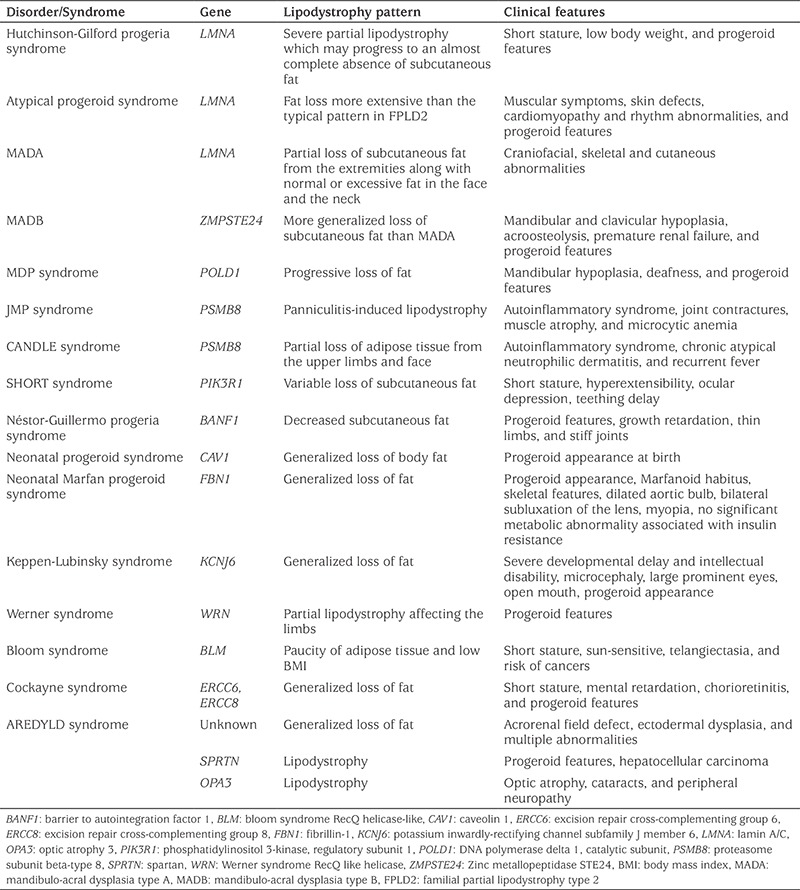
Progeroid disorders and other rare complex genetic disorders associated with lipodystrophy syndromes
